# Implementation of basic quality control tests for malaria medicines in Amazon Basin countries: results for the 2005–2010 period

**DOI:** 10.1186/1475-2875-11-202

**Published:** 2012-06-15

**Authors:** Victor S Pribluda, Adrian Barojas, Arletta Añez, Cecilia G López, Ruth Figueroa, Roxana Herrera, Gladys Nakao, Fernando HA Nogueira, Gerson A Pianetti, Marinete M Povoa, Giselle MR Viana, Margarete S Mendonça Gomes, Jose P Escobar, Olga L Muñoz Sierra, Susana P Rendon Norena, Raúl Veloz, Marcy Silva Bravo, Martha R Aldás, Alison HindsSemple, Marilyn Collins, Nicolas Ceron, Karanchand Krishnalall, Malti Adhin, Gustavo Bretas, Nelly Hernandez, Marjorie Mendoza, Abdelkrim Smine, Kennedy Chibwe, Patrick Lukulay, Lawrence Evans

**Affiliations:** 1 Promoting the Quality of Medicines Program, United States Pharmacopeia, Rockville, MD, 20852, USA; 2 Pan American Health Organization, La Paz, Bolivia; 3 Laboratorio de Control de Calidad de Medicamentos y Toxicología, Instituto Nacional de Laboratorios de Salud, La Paz, Bolivia; 4 Gerencia de Salud- Yacuiba, Tarija, Bolivia; 5 Servicio Departamental de Salud, Pando, Bolivia; 6 Gerencia De Salud, Riberalta, Beni, Bolivia; 7 Faculdade de Farmácia, Universidade Federal de Minas Gerais, Belo Horizonte, Minas Gerais, Brazil; 8 Laboratório de Pesquisas Básicas em Malária do Instituto Evandro Chagas, Pará, Brazil; 9 Laboratório de Controle da Qualidade de Medicamentos do Laboratório Central de Saúde Pública do Amapá, Amapá, Brazil; 10 Pan American Health Organization, Bogotá, Colombia; 11 Laboratorio Departamental de Salud Pública, Antioquia, Medellín, Colombia; 12 Servicio Nacional de Control de Enfermedades Transmitidas por Vectores Artrópodos, Quito, Ecuador; 13 Instituto Nacional de Higiene y Medicina Tropical “Leopoldo Izquieta Pérez”, Guayaquil, Ecuador; 14 Food and Drug Department, Ministry of Health, Georgetown, Guyana; 15 Pan American Health Organization, Georgetown, Guyana; 16 Chief Inspector, Vector Control Unit, Ministry of Health, Georgetown, Guyana; 17 Department of Medical Chemistry, Anton de Kom (ADEK) University of Suriname, Paramaribo, Suriname; 18 Pan American Health Organization, Quito, Ecuador; 19 Dirección General de Salud Ambiental, Ministerio del Poder Popular para la Salud, Caracas, Venezuela; 20 Consultant to the Promoting the Quality of Medicines Program, Casablanca, Morocco

## Abstract

**Background:**

Ensuring the quality of malaria medicines is crucial in working toward malaria control and eventual elimination. Unlike other validated tests that can assess all critical quality attributes, which is the standard for determining the quality of medicines, basic tests are significantly less expensive, faster, and require less skilled labour; yet, these tests provide reproducible data and information on several critical quality attributes, such as identity, purity, content, and disintegration. Visual and physical inspection also provides valuable information about the manufacturing and the labelling of medicines, and in many cases this inspection is sufficient to detect counterfeit medicines. The Promoting the Quality of Medicines (PQM) programme has provided technical assistance to Amazon Malaria Initiative (AMI) countries to implement the use of basic tests as a key screening mechanism to assess the quality of malaria medicines available to patients in decentralized regions.

**Methods:**

Trained personnel from the National Malaria Control Programmes (NMCPs), often in collaboration with country’s Official Medicine Control Laboratory (OMCL), developed country- specific protocols that encompassed sampling methods, sample analysis, and data reporting. Sampling sites were selected based on malaria burden, accessibility, and geographical location. Convenience sampling was performed and countries were recommended to store the sampled medicines under conditions that did not compromise their quality. Basic analytical tests, such as disintegration and thin layer chromatography (TLC), were performed utilizing a portable mini-laboratory.

**Results:**

Results were originally presented at regional meetings in a non-standardized format that lacked relevant medicines information. However, since 2008 information has been submitted utilizing a template specifically developed by PQM for that purpose. From 2005 to 2010, the quality of 1,663 malaria medicines from seven AMI countries was evaluated, mostly collected from the public sector, 1,445/1,663 (86.9%). Results indicate that 193/1,663 (11.6%) were found not to meet quality specifications. Most failures were reported during visual and physical inspection, 142/1663 (8.5%), and most of these were due to expired medicines, 118/142 (83.1%). Samples failing TLC accounted for 27/1,663 (1.6%) and those failing disintegration accounted for 24/1,663 (1.4%). Medicines quality failures decreased significantly during the last two years.

**Conclusions:**

Basic tests revealed that the quality of medicines in the public sector improved over the years, since the implementation of this type of quality monitoring programme in 2005. However, the lack of consistent confirmatory tests in the quality control (QC) laboratory, utilizing methods that can also evaluate additional quality attributes, could still mask quality issues. In the future, AMI countries should improve coordination with their health authorities and their QC lab consistently, to provide a more complete picture of malaria medicines quality and support the implementation of corrective actions. Facilities in the private and informal sectors also should be included when these sectors constitute an important source of medicines used by malaria patients.

## Background

The quality of medicines is a global problem and one of particular concern in developing countries, where limited human and financial resources may impair the implementation of appropriate quality assurance and quality control (QA/QC) systems. The impact that this may have on medicines utilized for the treatment of malaria, one of the major endemic diseases in the world, could be severe. Poor quality medicines have a detrimental effect on the lives of patients by causing them to remain ill longer and spend more time and financial resources obtaining treatment. They also can undermine a country’s health care system, exhaust financial and human resources, and exacerbate health problems, especially those associated with infectious disease, particularly the development of resistance. Several reports attest to the significance of this problem for malaria medicines in Africa and Asia [1-12]. On the other hand, published data for malaria medicines in the Americas is scarce [13,14].

In most countries, the institution responsible for ensuring the quality of medicines is the national Medicines Regulatory Authority (MRA), which performs this task by implementing Medicines Quality Monitoring (MQM) activities. To support these activities, it is crucial that the MRA have access to a laboratory that can properly perform QC analysis. Such testing is usually carried out by the Official Medicine Control Laboratory (OMCL), particularly for medicines used by Ministry of Health (MoH) programmes. All the countries included in this study had an assigned OMCL.

The MQM activities described in this report took place in the context of the Amazon Malaria Initiative (AMI) [15]. This initiative, established by the the United States Agency for International Development (USAID), was launched in 2001; it utilizes a holistic approach with the overall goal of preventing and controlling malaria and decreasing national morbidity and mortality in the region. AMI currently is implemented in seven countries in the Amazon Basin (Bolivia, Brazil, Colombia, Ecuador, Guyana, Peru, and Suriname) and selected countries in Central America; until 2008 Venezuela also participated in this initiative. AMI activities are carried out by six technical international partners with varied expertise, all of which work in close coordination with each other and with national counterpart stakeholders. The Promoting the Quality of Medicines (PQM) programme [16], and its predecessor the Drug Quality and Information (DQI) programme, implemented by the United States Pharmacopeia (USP), has been involved in AMI since 2002, providing technical assistance to ensure the quality of malaria medicines.

Several of the indicators suggested in the Medicine Quality Assessment Reporting Guidelines (MEDQUARG) [17] were an integral part of the assessments and are included in this report.

### Quality control of malaria medicines in AMI countries

Initial assessments performed by PQM identified several gaps associated with QA/QC systems in AMI countries: (a) There was no information on the quality of malaria medicines prior to distribution. Medicines in the public sector were assumed to be of good quality because they were distributed by the Ministry of Health; and although malaria medicines were provided free of charge at public health centres, patients could purchase malaria medicines outside of the public sector; (b) Storage conditions at different levels of the distribution chain were deficient, posing the risk of affecting the quality of the medicines administered to patients (see [14] and references within); (c) The capabilities of most of the OMCLs to perform full quality testing according to registration specifications was seriously impaired by lack of finances, personnel and equipment resources. In addition, most of the NMCPs in AMI countries were vertical, operating independently of the MRA and/or the OMCL. While addressing the strengthening of QA/QC systems in a more systematic manner, PQM recommended screening malaria medicines in AMI countries utilizing the three-level approach [18]. Each level in this approach consists of different QC procedures, which increase in complexity and complement the previous levels. Following is a brief description of each level:

Level 1 encompasses visual and physical inspection (V&P) to assess the physical characteristics of the medicines and compliance with specifications for two critical quality attributes: packaging, and labelling. The latter includes checking the expiration date and assessing the package and/or insert for fraudulent, incorrect, and /or deficient information. In addition, the physical inspection of dosage forms may provide useful information about the manufacturing processes (i.e., tablet preparations procedures resulting in cracked, chipped or broken tablets).

Level 2 comprises methodologies that can be performed in the field. Currently, as implemented by PQM, utilizes basic tests to evaluate four critical medicines quality attributes - identity, content, impurities, and disintegration. Identity, content, and impurities are assessed by Thin Layer Chromatography (TLC), comparing the presence and intensity of spots with those of the corresponding reference standards. Disintegration assesses the capability of solid oral dosage forms (tablets and capsules) to disperse in an aqueous environment within a preset period of time. Although disintegration is not a proxy for assessing dissolution, it is a prerequisite for proper dissolution to occur. Dissolution, which requires a more sophisticated methodology to be assessed, is the process required in order for the active pharmaceutical ingredient (API) to be dissolved and absorbed systemically.

Level 3 utilizes compendial tests or other validated methods to assess all quality attributes according to registration specifications. The critical attributes mentioned in Level 2 are evaluated typically with more advanced analytical techniques such as high-performance liquid chromatography (HPLC) and dissolution. While Level 1 and Level 2 may be assessed in the field by specially trained personnel and with minimal supplies, Level 3 requires skilled analysts and the infrastructure and equipment of an established laboratory.

According to PQM guidelines, Level 1 and Level 2 analysis are performed in the field on all samples, and failed samples, as well as a subset of passing samples, should be sent to the OMCL to perform Level 3 (confirmatory) testing. Alternatively, the OMCL may repeat Level 2 on these samples (verification tests) before submitting all or some of them to Level 3 testing. However, not all countries performed verification and/or confirmatory testing at the OMCLs; and in several countries the follow-up by OMCLs to field testing was not performed after every sampling round or at all sentinel sites. Therefore, this report focuses only on the results obtained by performing Level 1 and Level 2 testing by field personnel at sampling sites.

## Methods

The OMCL and field personnel from of each AMI country received training on sampling methodologies, performing V&P inspection and basic tests utilizing the Global Pharma Health Fund (GPHF) Minilab®, a portable kit containing the equipment, reference standards, and a manual of procedures to perform basic tests [19]. The manual provides TLC procedures for each medicine included in the Minilab®. For disintegration, the protocol requires tablets or capsules to be maintained in water for 30 minutes at room temperature and with occasional manual swirling. Confirmatory testing (Level 3) was carried out at the OMCL. In Brazil Artesunate was analyzed according to the International Pharmacopeia 4th edition, 2006; cloroquine, doxycycline, primaquine and quinine according to the United States Pharmacopeia USP29-NF24, and mefloquine according to a method developed and validated by the Faculdade de Farmácia, Universidade Federal de Minas Gerais. In Guyana chloroquine and quinine samples were analyzed according to the British Pharmacopiea BP 2010, and mefloquine according to the corresponding USP non-US monograph.

MQM activities were assumed in most countries by the NMCP, which developed a country- specific protocol to encompass sampling methods, sample analysis, and data reporting. The sampling sites were chosen based on malaria burden, accessibility, and geographical location. Sampling was performed throughout the year, and frequency varied amongst countries and within sites in each country. Sample collection was performed using convenience sampling, and countries were recommended to ensure proper storage of the sampled medicines to avoid compromising their quality after collection (e.g. maintaining samples in their original sealed containers according to the storage instructions for the respective product; packaging with suitable material to protect their integrity during transportation; ensuring correct sealing of containers; etc). Most medicines were sampled from the public sector (e.g., malaria diagnostic and treatment centers, hospitals), and seldom from the private sector (facilities with a license to sell medicines, e.g., pharmacies, retail stores) or the informal sector (facilities without a license to sell medicines, e.g., street markets, convenience stores, ambulatory vendors). In AMI countries, malaria medicines are usually procured centrally by the NMCP or other dependency within the MoH, and subsequently distributed to decentralized areas; modalities in these processes vary from country to country [20].

A sample was considered “failed” if it did not comply with V&P inspection and/or basic tests requirements, when performed as described above under Level 1 and Level 2. PQM recommends performing V&P inspection and basic tests on all samples except for expired medicines; however all expired medicines sampled in Bolivia and Colombia were also submitted to quality control testing. Samples were considered failed if they did not pass either basic test, disintegration or TLC; when failing one of them it was not submitted to the other.

## Results

This paper reports the QC results of malaria medicines sampled from 2005 to 2010 in AMI countries. All AMI countries have now adopted the use of basic tests as part of MQM at sentinel sites. However, due to logistic issues related in part to the management of certain reagents, Peru experienced delays in its implementation, and no results were received for the 2005–2010 period. Country-specific reports were initially developed by the NMCPs and, until 2007, the reporting format varied from country to country. Subsequently, PQM developed a uniform reporting format that was adopted by the countries and has been utilized since 2008.

### Sector of medicines sampled

The majority of the data reported by the NMCPs comes from medicines sampled in the public sector (1,445/1,663, 86.9%), as indicated in Table 1, with the private sector accounting for 152/1,663 (9.1%) and the informal sector for only 27/1,663 (1.6%). Most of the medicines sampled in the informal sector were from Guyana (13/27, 48.1%) and Suriname (11/27, 40.7%,), where malaria is endemic in the mining areas and public sector facilities are scarce and difficult to access. The informal sector is also known to exist in other countries, but no results from medicines in this sector were reported. The information of the sources of about 39/1,663 (2.3%) of the sampled medicines is unknown, because initial reports from certain countries did not identify the sector from which the medicines were sampled, particularly Ecuador (19/59, 32.2%,) in 2006 and Guyana (18/72, 25.0%,) in 2008.

**Table 1:** Country medicines sampled by sector

**Country**	**Year**	**Total Sampled**	**Sector**			
			**Public**	**Private**	**Informal**	**Unknown**
Bolivia	2006	139	139	0	0	0
	2007	76	66	10	0	0
	2008	67	67	0	0	0
	2009	24	24	0	0	0
**Bolivia Total**		**306**	**296 (96.7%)**	**10 (3.3%)**	**0**	0
Brazil	2006	32	28	4	0	0
	2007	187	179	4	3	1
	2008	94	94	0	0	0
**Brazil Total**		**313**	**301 (96.2%)**	**8 (2.6%)**	**3 (0.9%)**	**1 (0.3%)**
Colombia	2006	108	108	0	0	0
	2007	192	192	0	0	0
	2008	155	155	0	0	0
	2009	42	10	32	0	0
	2010	60	39	21	0	0
**Colombia Total**		**557**	**504 (90.5%)**	**53 (9.5%)**	**0**	**0**
Ecuador	2005	43	24	19	0	0
	2006	59	26	14	0	19
	2007	7	1	5	0	1
**Ecuador Total**		**109**	**51 (46.8%)**	**38 (34.9%)**	**0**	**20 (18.3%)**
Guyana	2007	62	57	0	5	0
	2008	72	37	9	8	18
	2009	33	30	3	0	0
	2010	122	96	26	0	0
**Guyana Total**		**289**	**220 (76.1%)**	**38 (13.1%)**	**13 (4.5%)**	**18 (6.2%)**
Suriname	2007	20	4	5	11	0
**Suriname Total**		**20**	**4 (20%)**	**5 (25%)**	**11 (55%)**	**0**
Venezuela	2006	12	12	0	0	0
	2007	57	57	0	0	0
**Venezuela Total**		**69**	**69 (100.0%)**	**0**	**0**	**0**
**Total**		**1663**	**1445 (86.9%)**	**152 (9.1%)**	**27 (1.6%)**	**39 (2.3%)**

### Quality control results

Table 2 summarizes the findings of V&P inspection and basic tests results reported by AMI countries. Except for two countries (Suriname and Venezuela), results for over 100 samples per country were submitted and since 2008 most indicated a significant decrease in the percentage that did not pass QC testing (failures). Of the results submitted, 193/1,663 (11.6%) of the medicines were classified as failing.

**Table 2:** Results of visual and physical inspection and basic tests by country

**Country**	**Year**	**Total Sampled**	**Total Failed**	**Failed V & P Inspection**^**1**^		**Failed Basic Tests**	
				**Total**	**Expired**	**Disintegration**	**TLC**
Bolivia^2^	2006	139	37	37	37	0	0
	2007	76	21	19	19	2	0
	2008	67	0	0	0	0	0
	2009	24	0	0	0	0	0
**Bolivia Total**		**306**	**58 (19.0%)**	**56 (18.3%)**	**56 (18.3%)**	**2 (0.7%)**	**0**
Brazil^3^	2006	32	7	4	4	0	3
	2007	187	12	1	1	0	11
	2008	94	1	1	1	0	0
**Brazil Total**		**313**	**20 (6.4%)**	**6 (1.9%)**	**6 (1.9%)**	**0**	**14 (4.5%)**
Colombia^2^	2006	108	0	0	0	0	0
	2007	192	17	10	1	7	0
	2008	155	29	29	29	0	0
	2009	42	0	0	0	0	0
	2010	60	0	0	0	0	0
**Colombia Total**		**557**	**46 (8.3%)**	**39 (7.0%)**	**30 (5.4%)**	**7 (1.3%)**	**0**
Ecuador	2005	43	11	6	4	1	4
	2006	59	7	6	6	1	0
	2007	7	0	0	0	0	0
**Ecuador Total**		**109**	**18 (16.5%)**	**12 (11.0%)**	**10 (9.2%)**	**2 (1.8%)**	**4 (3.7%)**
Guyana	2007	62	1	0	0	1	0
	2008	72	18	6	6	12	0
	2009	33	1	1	1	0	0
	2010	122	1	1	1	0	0
**Guyana Total**		**289**	**21 (7.3%)**	**8 (2.8%)**	**8 (2.8%)**	**13 (4.5%)**	**0**
Suriname	2007	20	0	0	0	0	0
**Suriname Total**		**20**	**0**	**0**	**0**	**0**	**0**
Venezuela	2006	12	4	4	0	0	0
	2007	57	26	17	8	0	9 (13.0%)
**Venezuela Total**		**69**	**30 (43.5%)**	**21 (30.4%)**	**8 (11.6%)**	**0**	**9 (13.0%)**
**Total**		**1663**	**193 (11.6%)**	**142 (8.5%)**	**118 (7.1)**	**24 (1.4%)**	**27 (1.6%)**

V&P = visual and physical inspection; TLC = thin-layer chromatography.

^**1**^ In the V&P columns, the first represent total failures, including expired samples, and the second only expired samples.

^**2**^ All expired samples were submitted to quality control testing.

^**3**^ For doxycycline, (Brazil) and dihydroartemisinin or piperaquine (Suriname), there was no validated TLC method available at the time and only disintegration was assessed.

It is worth noting that from the 11.6% total failure rate, 8.5% (142/1663, 8.5%) was due to failure in V&P inspection. This trend, in which the majority of failures are due to V&P inspection, was observed in four of the seven countries assessed. Finally, as indicated in Table 2, all the samples failing V&P inspection in Bolivia (56), Brazil (6), and Guyana (8) failed because they were expired when collected, as did most of the samples in Colombia (30/39) and Ecuador (10/12).

Overall, expired medicines accounted for 118/142 (83.1%) of the samples that failed V&P inspection. The two countries experiencing the highest rate of expired products (Bolivia in 2006 and 2007, and Colombia in 2008) addressed this problem by better controlling their inventory, and in subsequent years no expired medicines were reported.

The two countries in which the majority of V&P inspection failures were not due to expired medicines were Colombia in 2007 and Venezuela in 2006 and 2007. In Colombia, most of these failures were due to damaged packages, in which blisters were not tightly sealed or the legend was illegible. In Venezuela, sampled medicines were not in the original package and lacked information on their source, lot number and/or expirations dates; also in Venezuela V&P inspection identified samples containing irregularly shaped, chipped or broken tablets. Disintegration was the least failed basic test with 24 failures (1.4%), a few more samples, 27 (1.6%), failed TLC. In four countries (Bolivia, Colombia, Ecuador, and Venezuela) V&P inspection accounted for the majority of failures; in Brazil and Guyana failures in basic tests were more prominent. A single medicine accounted for all the failures in basic tests in Colombia (seven sulphadoxine-pyrimethamine samples), Guyana (13 chloroquine samples) and Brazil (14 quinine samples).

Following is a brief summary of findings per country that includes the type of medicines failing basic tests:** Bolivia.** Data for a total of 306 samples were submitted from Bolivia between 2006 and 2009. Most failures (56/58) were due to expired medicines; however, in subsequent years, as mentioned above, no expired medicines were identified. The only failures in basic tests detected in Bolivia (2007) were 2/30 (6.7%) chloroquine samples that did not pass disintegration test. Even though PQM recommended not to submit expired samples to quality control testing, the large number of expired samples were analyzed; no failures were detected amongst those.** Brazil.** Data for a total of 313 samples were submitted from Brazil between 2006 and 2008. The 14 failures in basic tests were sampled that failed the TLC test, with quinine accounting for 3/5 (60.0%) in 2006, and 11/41 (26.8%) in 2007. Seven quinine samples that failed TLC were subsequently analysed at the reference laboratory and passed compendial (confirmatory) testing; however, only field results are included in Table 2. After 2008, Brazil NMCP discontinued the use of basic tests for the assessment of malaria medicines quality, while evaluating alternative processes to perform QC of malaria medicines. **Colombia.** Data for a total of 557 samples were submitted from Colombia between 2006 and 2010. The number of failed medicines decreased significantly during the last two years assessed, with no failures reported in 2009 and 2010. The overall failure rate was 8.3% (46/557), mostly due to V&P inspection (39/46), of which 30 samples were expired medicines. In Table 2, among all the samples that failed in 2007, 7/23 (30.4%), were sulphadoxine-pyrimethamine containing medicines that failed disintegration. Even though PQM recommended not to submit expired samples to quality control testing, the large number of expired samples were analyzed, but no failures were detected. **Ecuador.** Data for a total of 109 samples were submitted from Ecuador between 2005 and 2009. No reports were received for 2007 and 2008. The data indicates a drop in the failure rate over time as well. All the failures were found in 2005 (n: 11) and 2006 (n: 7); 10 of 12 samples failing V& P inspection were expired medicines. In 2005, 4/11 (36.4%) primaquine samples failed TLC and 1/8 (12.5%) sulphadoxine-pyrimethamine containing medicine failed disintegration. In 2006, 1/2 (50.0%) quinine sample failed disintegration. **Guyana.** Data for a total of 289 samples were submitted from Guyana between 2007 and 2010. Of the 21 samples that failed over four years, 18 were identified in 2008. All those failing V&P inspection (n: 8) were expired medicines, while 1/14 (7.1%) and 12/12 (100%) chloroquine samples failed disintegration in 2007 and 2008 respectively. **Suriname.** Data was submitted from Suriname only in 2007, and it comprises 20 samples. From these, one sample contained dihydroartemisinin and 11 consisted of Artecom®^,^ a fixed-dose combination (FDC) therapy reported to contain dihydroartemisinin, piperaquine and trimethoprim, for which there was no basic test available at the time. No information was provided on whether the blisters with the FDC in the Artecom® samples contained also a single primaquine tablet (see below). The results in Table 2 reflect only the disintegration test assessed for those 12 samples. Based on this and the small number of medicines collected, no definite conclusion may be drawn from this country data. It is worth noting that a FDC with the same brand name (Artecom®) was the most prevalent malaria medicine identified in a recent study performed in the private and informal market in Guyana and Suriname, in which each blister in the package contained a single primaquine tablet [21]. All the samples of this medicine in the mentioned studies failed V&P inspection because there was no content information on the dose of the primaquine tablet in the package or the insert.** Venezuela.** A total of 69 medicines were reported by Venezuela, in 2006 and 2007, with a failure rate of 43.5% (30/69). However, as was the case of other countries, most of these failures were due to V&P inspection (21/30, 70%). During 2007 TLC failures were found in 4/17 (23.5%) cloroquine samples, 2/13 (15.4%) mefloquine samples and 3/24 (12.5%) primaquine samples. Venezuela ceased participating in AMI activities in 2008.

Table 3 provides information according to the API of the sampled medicines, which supplements the data detailed above for the medicines that failed in each country. Primaquine (n: 455) and chloroquine (n: 447) were the most often sampled and analysed medicines representing 54.2% of the total, while artesunate and other medicines containing artemisinin-derivatives accounted for 19.2% (n: 320). Taking into account that the majority of medicines were sampled in the public sector, the larger numbers for chloroquine and primaquine probably reflect the fact that *Plasmodium vivax* is the prevalent malaria infection found in the region, and these two medicines are included in the national and World Health Organization (WHO) guidelines for its treatment [22]. It is important to note, that all the failures for artemisinin-derivative medicines (n: 22) were due to V&P inspection. Medicines containing sulphadoxine-pyrimethamine had the highest rate of failure at 20.9% (14/67), of which six failed V&P inspection and eight failed disintegration. It is interesting to note that dissolution was the most common reason for sulphadoxine- pyrimethamine failures reported in other studies [7,8,23,24]. Whether in this case there was any correlation between these two quality attributes cannot be assessed because of the lack of confirmatory testing for dissolution at the laboratory. Table 4 shows results from a subset of samples that underwent both basic and confirmatory testing in Brazil and Guyana. The data attests to the importance of performing confirmatory testing on subsets of the samples, including a percentage of those that pass basic tests. In Brazil, 7 quinine samples that failed basic tests were found to be compliant by confirmatory tests. It is worth noting that in Brazil, 3 other quinine samples with the same lot number of those included in Table 4, were collected at another sentinel site and passed both basic test and confirmatory testing (data not shown). On the other hand, in Guyana, five samples (one chloroquine and four quinine) that passed TLC, failed the confirmatory testing for content of API. The compendial method that assesses content revealed that those five samples were outside the label specifications; though those differences were too small to be detected through TLC.

**Table 3:** Test Results by API in Medicine

**Active Pharmaceutical Ingredientq**	**Failures**
Amodiaquine	1/49 (2.0%)
Artemether^1^	2/10 (20.0%)
Artemether and Lumefantrine^1^	10/209 (4.8%)
Artesunate^1^	10/89 (11.2%)
Chloroquine	66/447 (14.8%)
Dihydroartemisinin^3^	0/1 (0%)
Dihydroartemisinin-Piperaquine- Trimethoprim^2^	0/11 (0%)
Doxycycline^1, 2^	2/60 (3.3%)
Mefloquine	16/99 (16.2%)
Primaquine	55/455 (12.1%)
Quinine	22/130 (16.9%)
Sulfadoxine-Pyrimethamine	14/67 (20.9%)
Tetracycline	0/6 (0%)

^1^ Artemisinin derivatives containing medicines and doxycycline samples failed only V&P Inspection.

^2^ For medicines containing doxycycline, in Brazil, and dihydroartemisinin or piperaquine, in Suriname, there was no validated TLC method available at the time; therefore, only disintegration was assessed. Subsets of doxycycline samples sent to the reference laboratory in Brazil passed validated tests for identification and content.

**Table 4:** Comparison of Basic and Confirmatory Test Results

**Country**	**API**^**1**^	**Basic Tests**		**Confirmatory Testing for Content**^**2**^	
		**Pass**	**Fail**	**Pass**	**Fail**
Brazil	Artesunate	1	0	1	0
	Chloroquine	1	0	1	0
	Doxycycline^3^	2	0	2	0
	Mefloquine	1	0	1	0
	Primaquine	5	0	5	0
	Quinine	0	7	7	0
Guyana	Chloroquine	13	0	3	1
	Mefloquine	1	0	1	0
	Quinine	14	0	1	4
**Total**		**38**	**7**	**22**	**5**

^1^ Medicines collected during one round of sampling at one sentinel site in each country.

^2^ All samples passed the identity test.

^3^ There was no validated TLC method available.

## Discussion

The overall failure rate found for samples collected during the 2005–2010 survey (11.6%) is significantly lower than that reported in other regions of the world [1-10]. It is important to keep in mind that the scope of most of the other studies, the number of medicines collected, the sectors from which the medicines were sampled, and the methodologies utilized were different; all of which precludes drawing major conclusions from a comparison of overall failures rates. Among those studies, two of those performed in Africa [7,8] merit particular attention because both basic tests and compendial tests were utilized to assess subsets of the sampled medicines.

The followings sections describe positive outcomes from the implementation of basic QC tests by NMCPs in AMI countries:

### Unique regional QC approach in Latin America and the Caribbean (LAC)

Despite the differences in their QA/QC systems, the approach discussed in this paper was embraced by all participating AMI countries; this allowed regional collaborations and exchange of experiences throughout the years and provided the first documented regional information on the quality of malaria medicines. There is no other programme in LAC pursuing a regional approach to QC for other endemic diseases, including other vector-borne diseases.

### Feasibility to perform QC of medicines in remote areas

In certain countries, the incidence of malaria is most prevalent in remote areas where access is difficult due to geographical barriers and the cost associated with transport; particularly in gold mining areas in Brazil, Guyana and Suriname. Storage conditions in these regions are frequently deficient and public services are difficult to access; therefore there is a high risk of medicines deteriorating (see [14] and references within) and also of patients accessing medicines of questionable quality in the informal sector. Through this approach, data on the quality of medicines available to patients in these remote areas was gathered for the first time.

### Improvement in the quality of malaria medicines

Overall, the results of this report clearly indicate that the rate of malaria medicines failures in AMI countries compares favorably with other regions of the world and these failures have decreased significantly in recent years, at least in the public sector. The very small number of failures identified during the last two years (see Table 2) contrast dramatically with the results of the studies performed in Africa [7,8], in which sampling was performed just one year before. The following aspects are worth highlighting: a. The majority of failures were due to V&P inspection, most of which were expired medicines; their number decreased dramatically in all countries after 2008. Medicines’ shelf life is based on manufacturer’s stability studies, which ensure that medicines properly stored during the period of validity maintain their quality attributes. Medicines that are of good quality when procured may degrade and become of poor quality if not stored properly (see [14] and references within) or if stored beyond their expiry date. If expired medicines are analyzed and found non-compliant with their quality specifications, it could be more difficult to implement the proper corrective action with the manufacturer because degradation of an otherwise good quality medicine could be the cause. See [17] and references within for definitions and distinctions between counterfeit, substandard and degraded products. Based on V&P inspection or basic tests results it was not obvious that the poor quality medicines identified were counterfeits. With the type of failures identified, determining whether they were due to counterfeits would have required confirmatory and/or other forensic testing and follow up with the manufacturer. b. Medicines containing artemisinin derivatives failed “only” V&P inspection. In contrast, the two recent studies in Africa [7,8] identified failures in artemisinin-containing medicines in samples submitted to both basic tests and undergoing compendial testing. Poor quality artemisinin derivatives could be a factor in generating resistance to a family of drugs that has no replacement in the foreseeable future for the treatment of falciparum malaria. This is of particular relevance because of recent reports suggesting a rise in resistance to artemisinine derivatives *in vivo* and *in vitro* in southeast Asia [25,26], a region in which poor quality samples of these products have been identified. MQM activities in AMI countries using basic tests as a screening methodology also faced three main challenges, described in more detail below.

### Lack of verification/confirmatory testing at the laboratory of samples tested in the field

A critical component of conducting MQM activities utilizing the three-level approach is to perform verification tests (repetition of basic tests) and/or confirmatory tests (testing according to compendial or other validated methods utilized for registration) at the OMCL, on subsets of the samples tested in the field. However, since a very limited number of samples underwent these tests, caution needs to be exerted when drawing conclusions on the overall rate of failures reported utilizing basic tests in the field.

The cases of the mefloquine and quinine samples in Guyana that passed basic tests in the field but failed at the OMCL, and the quinine samples in Brazil, which failed in the field but passed at the reference laboratory, attests to the need of support from a laboratory (see Table 4). The more sensitive methods utilized to assess content allows for the detection of small differences in the levels of APIs and presence of impurities. The different results on the quinine samples from Brazil that failed at one site and were compliant at another highlight the convenience of the laboratory performing occassionally verification (basic) tests to assess the proficiency of field personnel.

The additional information that compendial testing can provide, also in subsets of samples passing basic tests, is further exemplified in the two studies performed in Africa [7,8]. In those, the results for samples submitted to basic and/or compendial testing showed a significantly higher rate of failures in the latter. This tendency was due in part to the type of analysis (disintegration vs. dissolution) and the sensitivity of the methodology (particularly in the case of impurities for artemisinin derivatives).

Not performing verification and/or confirmatory testing as a regular practice has been the primary deficiency in the management of MQM activities by NMCPs in most AMI countries. Although timely delivery of results by the lab was a problem, more often than not, the primary issue was the lack of coordination between NMCPs, MRAs and OMCLs while performing MQM activities at sentinel sites.

### Sampling primarily from the public sector

Most medicines tested were sampled in the public sector (86.9%). As a result, the current MQM data may not necessarily reflect the entire market available to patients, particularly in areas where malaria medicines are purchased in the private and informal sectors due to scarcity of public sector facilities and/or difficulties in accessing those facilities. Recent case studies performed in the private and informal sectors in Guyana and Suriname [21], where the percentage of failed samples was significantly higher than those reported in this article confirm this assumption. However, in those case studies medicines were analysed by compendial methods, and the higher percentage of failed samples partially reflects this fact. In addition, in both countries all Artecom® samples failed because of the reasons detailed under Suriname in the Results section. Similarly, in one of the recent studies performed in Africa [7], the failure rates in the private and informal sectors were 1.8 and 2.5 higher than in the public sector.

### Inability to test all available malaria medicines due to the lack of basic tests procedures

Doxycycline medicines in Brazil and more than 50% of the samples (Artecom®) from Suriname could not be assessed by TLC due to the lack, at the time, of a validated TLC methodology.

### General considerations on the use of basic tests

Basic tests offer a consistent, rapid, and cost-effective means for monitoring medicines quality in the field. Those are of the utmost importance for these countries’ needs.

(a) In endemic areas, where medicines are in constant demand but storage conditions are frequently deficient, it enables constant surveillance in a timely manner. (b) Many endemic areas, such as gold mining regions in Brazil, Guyana and Suriname, are very difficult to access. The minimal equipment and materials required for basic tests enables them to be performed *in situ,* obviating the needs of time consuming, often expensive and difficult shipment to the OMCL. (c) In many AMI countries, local health offices in decentralized areas usually operate under stringent budgets that limit both the personnel available to perform tests and the availability of costly reagents and equipment; the use of basic tests provides a cost-effective means to address these circumstances.

It is very important, however, to also take into account the limitations of this approach:

(a) The Guyana 2010 results show that basic tests, specifically TLC, found fewer failures than the confirmatory tests used to analyse the same medicines. The assay value obtained for chloroquine with the TLC confirmatory test was 90.0%, just outside the range (93.0% to 107.0%) of the HPLC confirmatory test method. The four quinine medicines that failed the HPLC confirmatory testing did so by having averages ranging from 106.3% to 111.5%. The fractional differences found at the OMCL could hardly be detected using the semi-quantitative visual assessment of the intensity of the TLC spots. In addition, visual detection of spots in TLC is subjective and may also lack the sensitivity to identify certain impurities; a problem frequently encountered for artemisinin derivatives. Even if small deviations are detected with basic tests, it is necessary to perform confirmatory testing; but proper judgement needs to be exerted on their use when urgent treatement is required. (b) The Brazil 2007 results for the quinine samples that failed TLC testing, uncover another limitation of basic tests performed in the field: poor field conditions or poorly trained personnel. The tests performed on the same medicines at the lab indicated that those samples passed compendial testing, supporting the recommendation of sending all failed samples for verification and/or confirmatory testing. (c) Despite the fact that poor disintegration could impact negatively on the dissolution rate, it is important to reiterate that disintegration is not a proxy for dissolution. This must be taken into account even when considering that all the sulphadoxine-pyrimethamine that failed basic tests, 57.1% (8/14) failed disintegration, though dissolution has been a common problem for sulphadoxine-pyrimethamine in other studies [6,7,16,17].

Regardless of the benefits that basic tests offer, it is equally important to understand their limitations, a conclusion also drawn from the recent studies in Africa [7,8]. These limitations need to be considered when comparing basic tests with compendial testing for certain quality attributes, such as dissolution and impurities [7,8] or with more elaborated methodologies, such as Raman and/or near-infrared spectrometry [27,28]. The limitations, on the other hand, emphasize the need of utilizing basic tests in a context of periodic assessment also by compendial methods.

## Conclusions

According to V&P inspection and basic test results, the quality of malaria medicines in AMI, primarily in the public sector, has improved over the years since the implementation of this type of assessment in 2005. Most failures were due to medicines that did not pass V&P inspection, and the majority of those were expired medicines. Only 3% of the samples failed basic analytical tests, which compares very favorably with results obtained recently in Africa and Southeast Asia. However, AMI countries need to perform confirmatory testing more consistently in order to strengthen their quality assurance systems for malaria medicines. Due to the lack of confirmatory testing, quality issues such as dissolution and/or impurities may have passed unnoticed and/or may still persist.

The benefits of periodic and systematic assessment as a diagnostic tool in the supply chain is well exemplified in the results of the two countries showing the greatest number of expired medicines in early years, which was subsequently corrected.

There is also a need to assess more frequently the private and informal sectors, particularly in areas where patients do not have access to the medicines distributed by the NMCP. Having evidence on the availability of malaria medicines in these sectors and their quality will help health authorities implement the appropriate corrective actions.

A better coordination of NMCP activities with MRAs and OMCLs needs to be implemented. This will allow not only the withdrawal of failed medicines from storage facilities and/or points of use, but also implementation of proper follow-up on the root cause of the problem along the supply chain, whether it be due to poor QA/QC during procurement, manufacturers not complying with GMP, deficiencies in distribution practices, or poor management of inventories. The implementation of V&P inspection and basic tests by NMCP provided much needed data on the quality of malaria medicines in AMI countries, mostly in the public sector. Although this has been a very important first step in ensuring that patients receive quality-assured medicines, there is room for improvement in this process, mainly by the involvement of all the relevant stakeholders.

## Abbreviations

AMI, Amazon Malaria Initiative; API, Active Pharmaceutical Ingredient; DQI, Drug Quality and Information Programme (USP); FDC, Fixed-dose combination; GMP, Good Manufacturing Practices; GPHF, Global Pharma Heath Fund; HPLC, High performance liquid chromatography; LAC, Latin America and the Caribbean; MQM, Medicine Quality Monitoring; MRA, Medicines Regulatory Authority; NMCP, National Malaria Control Programme; OMCL, Official Medicines Control Laboratory; PQM, Promoting the Quality of Medicines Programme; QA, Quality Assurance; QC, Quality Control; TLC, Thin Layer Chromatography; USAID, United States Agency for International Development; USP, United States Pharmacopeia; V&P, Visual and Physical Inspection; WHO, World Health Organization..

## Competing interests

The authors declare that they have no competing interests.

## Authors’ contributions

AA, CGL, RF, RH and GN were involved in MQM activities in Bolivia. FHAN, GAP, MMP, GMRV and MSMG were involved in MQM activities in Brazil. JPE, OLMS and SPRN were involved in MQM activities in Colombia. RV, MSB and MRA were involved in MQM activities in Ecuador. AHT, MC, NC and KK were involved in MQM activities in Guyana. MA and GB were involved in MQM activities in Suriname. NH and MM were involved in MQM activities in Venezuela. AB, AS and VSP provided guidance to NMCPs and reviewed data from sentinel sites. LE and VSP performed data analysis and drafted the manuscript with additional input from AS, KC and PL. All authors read and approved the final manuscript.
